# Changes in patient characteristics and procedural measures over a 10-year period in aortic valve-in-valve procedures for degenerated surgical bioprostheses

**DOI:** 10.3389/fcvm.2025.1680733

**Published:** 2025-11-10

**Authors:** Tim Knochenhauer, Till J. Demal, Oliver D. Bhadra, Sebastian Ludwig, Nils Arne Sörensen, Ina von der Heide, Laura Hannen, David Grundmann, Lisa Voigtländer-Buschmann, Lara Waldschmidt, Johannes Schirmer, Simon Pecha, Evaldas Girdauskas, Stefan Blankenberg, Hermann Reichenspurner, Niklas Schofer, Andreas Schaefer

**Affiliations:** 1 Department of Cardiovascular Surgery, University Heart and Vascular Center Hamburg, Hamburg, Germany; 2 DZHK (German Center for Cardiovascular Research), Partner Site Hamburg/Kiel/Lübeck, Hamburg, Germany; 3 Department of Cardiology, University Heart and Vascular Center Hamburg, Hamburg, Germany; 4 Department of Cardiothoracic Surgery, Augsburg University Medical Center, Augsburg, Germany

**Keywords:** TAVI, TAVR, valve-in-valve, aortic valve, bioprosthesis, lifetime management

## Abstract

**Objectives:**

Aortic valve-in-valve procedures for treatment of degenerated surgical bioprostheses are an established therapy. In this study, we evaluated how the risk profiles, procedural approaches, and early outcomes for patients in these procedures changed over a period of 10 years.

**Methods:**

Baseline, procedural, early outcome, and echocardiographic parameters were retrospectively compared between three time periods (period 1: 2013–2016, period 2: 2017–2020, and period 3: 2021–2023).

**Results:**

Between 2013 and 2023, a total of 256 patients underwent valve-in-valve implantation in degenerated aortic bioprostheses at our center with a steady increase of patient numbers. The median age of the patients was 78.0 (interquartile range 72.2–82.4) years and remained unchanged over time. EuroSCORE II presented lower risk profiles in later periods (*p* = 0.001). Access proportions changed with transfemoral access in 100% of patients in period 3 (*p* < 0.001). Rates of BASILICA procedures (0% vs. 17.5% vs. 19.4%; *p* < 0.001) and valve fracturing steadily increased (0% vs. 6.3% vs. 7.8%; *p* = 0.058). Cerebral protection device use presented a distinct decline to 18.4% in period 3 (*p* < 0.001). Procedure time and length of intensive care unit stay decreased significantly over time. Early outcome parameters such as rates of permanent pacemaker implantation, bleeding, acute kidney injury, disabling stroke (0.0% vs. 1.3% vs. 1.0%; *p* = 0.653), and device success (91.8% vs. 92.5% vs. 98.1%; *p* = 0.123) showed no significant changes over time. The rate of 30-day mortality decreased to 0% in period 3 (*p* = 0.069).

**Conclusion:**

Advancements in technical approaches have expanded eligibility for patients previously considered unsuitable for aortic valve-in-valve procedures. In this study, it was found that early outcomes for patients were excellent, with improvement over time, highlighting the clinical efficacy and safety of the procedures.

## Graphical Abstract

**Figure:**
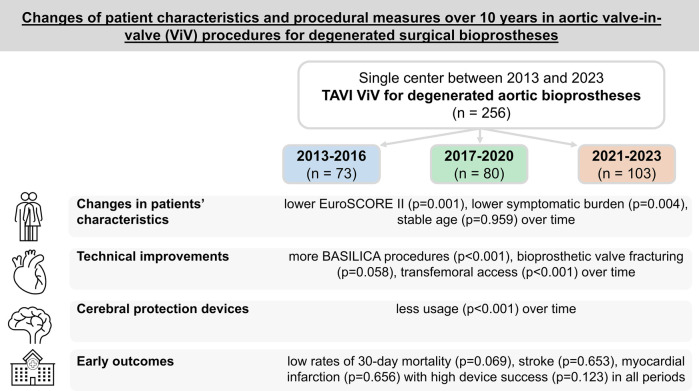
This single-center study investigates changes in patient characteristics and procedural measures over a period of 10 years in aortic valve-in-valve procedures for treatment of degenerated surgical bioprostheses. Baseline, procedural, early outcome, and echocardiographic parameters were retrospectively compared between three time periods (period 1: 2013–2016, period 2: 2017–2020, and period 3: 2021–2023). Main changes in characteristics, technical improvements, and early outcomes in patients are illustrated.

## Introduction

Aortic valve-in-valve (ViV) procedures for treatment of degenerated surgical bioprostheses are an established therapy. Because of technical improvements in transcatheter heart valves (THVs), increasing operator experience, and development of measures addressing unfavorable anatomies, ViV procedures have been increasingly performed over the last decade, even in patients previously considered not suitable for such procedures.

Unfavorable anatomies in aortic ViV procedures consist of low coronary ostia distance from the sewing ring of the index prosthesis with consecutive higher risk of coronary obstruction, especially in surgical bioprostheses with externally mounted leaflets, small bioprostheses (diameter ≤ 23 mm) (1) with anticipated detrimental hemodynamics after ViV in terms of elevated transvalvular pressure gradients or even patient–prosthesis mismatch, and hostile anatomies of iliac vessels in terms of severe calcification and/or tortuosity, a known risk factor for vascular complications and adverse outcomes following transcatheter aortic valve implantation (TAVI) for treatment of severe symptomatic native aortic valve stenosis.

To treat those patients at particularly high risk for periprocedural complications during the performance of ViV, several procedural refinements and changes in procedural measures have been implemented over the last decade: Bioprosthetic or native aortic scallop intentional laceration to prevent iatrogenic coronary artery obstruction (BASILICA) was introduced in 2019 (2) to prevent coronary obstruction, which is associated with a mortality rate of up to 50% (3). Furthermore, bioprosthetic valve fracturing (BVF) and/or utilization of supraannular valves with a rather high implantation height was found to potentially avoid elevated postprocedural transvalvular pressure gradients/patient–prosthesis mismatch after ViV with consecutive early degeneration of implanted THV (4–6). Improvements in percutaneous vascular closure systems, as well as reduction of THV sheath sizes for transfemoral access, result in reduced vascular complication rates, which is of particular importance, because major vascular complications lead to prolonged hospital stay, higher mortality, as well as higher rates of bleeding complications, access site infections, and renal impairment (7–9).

To assess the influence of the described new techniques for aortic ViV procedures and illustrate the changing patient profiles and utilized periprocedural measures in a special subset of patients, we herein evaluate all aortic ViV procedures over a period of 10 years at our center.

## Materials and methods

### Patient cohort and study design

All patients who underwent TAVI in degenerated aortic bioprostheses as a ViV procedure at our center during the period between 2013 and 2023 were included in this analysis.

To determine changes in the risk profiles, procedural data, and outcomes of patients over time, the cohort was divided into three subgroups based on the date of procedure (period 1: 2013–2016, period 2: 2017–2020, and period 3: 2021–2023).

### Study procedure

Institutional standards for aortic ViV procedures have been described previously (10). In this study, access routes were planned and executed based on multislice computed tomography examination. Transfemoral access with local anesthesia was the first-line approach when possible. Patients in whom intentional leaflet laceration was performed, general anesthesia was used to enable transesophageal echocardiography guidance. Utilized vascular closure systems consisted of suture-based devices (ProGlide/ProStyle/ProStar; Abbott, Abbott Park, IL, USA) or a collagen plug-based device (MANTA; Teleflex, Wayne, PA, USA). In recent years, all those patients in whom intentional leaflet laceration was not performed were provided with single femoral puncture as interventional access, and non-interventional access was gained via the right-sided radial artery. The target height for THV valve deployment was alignment of both lower stent rims to create optimal postinterventional hemodynamics with full stent deployment of the THV. Intentional leaflet laceration was performed as previously described (11). Bioprosthetic valve fracture (BVF) was performed according to the discretion of the surgeons and in recent years after THV implantation. The application of cerebral embolic protection was left to operator discretion and consisted of the Sentinel device (Boston Scientific, Marlborough, MA, USA). In recent years, all patients were postoperatively transferred to a holding area until the first postoperative day and the stay until discharge was completed in the ward (10).

### Statistical analysis

Outcome parameters were adjudicated in accordance with the updated standardized Valve Academic Research Consortium-3 (VARC-3) definitions (12).

Continuous variables were reported as medians with interquartile range (IQR; 25th percentile and 75th percentile) and compared using the Kruskal–Wallis test. Binary variables were shown as counts and frequencies and compared using the *χ*^2^ test.

Normality of continuous data deviation was tested by using the Shapiro–Wilk test.

Parameters predicting the VARC-3 composite endpoint device success were identified using logistic regression analysis. The parameters included in the model were the time period of the procedure, the femoral access route, the choice of THV type, cerebral protection, preballooning, postballooning, and the BASILICA procedure. Odds ratios, 95% confidence intervals (CI), and *p*-values were reported from this model.

For binary outcome parameters (including 30-day mortality, myocardial infarction, disabling stroke, acute kidney injury stage II or III, permanent pacemaker implantation, major vascular complications, type 3 or 4 bleeding, and device success), proportions were calculated for each study period. Exact 95% CIs were derived using binomial tests to provide reliable estimates of event rates independent of sample size distribution.

A *p*-value of <0.05 was considered statistically significant. All statistical analyses were performed using the statistical software RStudio 2023.12.1 (R Foundation for Statistical Computing, Vienna, Austria). Graphs were created using GraphPad Prism, version 9.5.1 (GraphPad Software, San Diego, CA, USA).

## Results

### Study population

Between 2013 and 2023, a total of 256 patients underwent TAVI as a ViV procedure in degenerated bioprostheses at our center. Of these, *n* = 73 were assigned to the period 1 group (2013–2016), *n* = 80 to the period 2 group (2017–2020), and *n* = 103 to the period 3 group (2021–2023).

### Baseline characteristics

The median age of the patients was 78.0 (IQR 72.2–82.4) years with a range of 48.3–96.2 years without any differences between the three groups. Risk stratification using EuroSCORE II presented a decrease of perioperative risk from 11.8% (IQR 6.0–15.5) in period 1 to 6.4% (IQR 2.8–16.0) in period 3 (*p* = 0.001). Patients in later periods presented with a lower symptomatic burden at the time of ViV as represented by lower percentages of New York Heart Association (NYHA) functional classes III or IV (89.0% vs. 77.5% vs. 67.3%; *p* = 0.004).

The durability of index surgical bioprostheses increased significantly over time [8.0 (IQR 6.0–11.0) vs. 11.0 (IQR 8.0–14.3) vs. 12.0 (IQR 9.0–14.0) years; *p* < 0.001]. Accordingly, a commonly considered early deteriorating surgical bioprosthesis with externally mounted leaflets presented the highest proportion as index valve in period 1 (34.2% vs. 23.8% vs. 7.8%; *p* < 0.001). An increase of deteriorated Perceval sutureless aortic valves (LivaNova, London, United Kingdom) treated by ViV was seen over time (0% vs. 1.3% vs. 8.7%; *p* = 0.004). Overall, 20.7% of patients had a surgical valve size of ≤21 mm, while in 36.7% of surgical valves, the true inner diameter measured ≤19 mm. The ViV procedure was performed in 49.6% of patients because of a structural valve deterioration of the surgically implanted aortic bioprostheses, which presented as a combined lesion with at least moderate stenosis and a concomitant moderate regurgitation component.

Detailed baseline characteristics are reported in Table 1.

**Table 1:** Baseline characteristics.

Variable	All (*n* = 256)	2013–2016 (*n* = 73)	2017–2020 (*n* = 80)	2021–2023 (*n* = 103)	*p*-Value
Age (years), median (IQR)	78.0 (72.2–82.4)	78.5 (72.7–83.0)	77.8 (73.5–82.4)	77.6 (72.0–82.2)	0.959
Age (years), range	48.3–96.2	51.1–89.8	50.7–90.1	48.3–96.2	
Male sex, *n* (%)	149 (58.4)	35 (47.9)	52 (65.0)	62 (60.2)	0.115
BMI (kg/m^2^), median (IQR)	26.5 (23.7–30.0)	27.3 (23.7–30.1)	26.5 (24.6–29.8)	26.1 (23.3–29.7)	0.656
EuroSCORE II (%), median (IQR)	8.1 (4.9–14.5)	11.8 (6.0–15.5)	6.3 (4.0–9.9)	6.4 (2.8–16.0)	0.001
Severely reduced ejection fraction <30%, *n* (%)	22 (8.6)	6 (8.2)	8 (10.0)	7 (6.8)	0.736
Arterial hypertension, *n* (%)	207 (80.9)	59 (80.8)	65 (81.3)	83 (80.6)	0.993
Insulin-dependent diabetes mellitus, *n* (%)	20 (7.8)	6 (8.2)	5 (6.3)	9 (8.7)	0.814
Coronary artery disease, *n* (%)	139 (54.3)	48 (65.8)	38 (47.5)	53 (51.5)	0.058
Prior stroke, *n* (%)	32 (12.5)	14 (19.2)	8 (10.0)	10 (9.7)	0.124
Prior myocardial infarction, *n* (%)	23 (9.0)	7 (9.6)	6 (7.5)	10 (9.7)	0.855
Chronic lung disease, *n* (%)	28 (10.9)	13 (17.8)	8 (10.0)	7 (6.8)	0.066
Creatinine (mg/dL), median (IQR)	1.17 (0.94–1.50)	1.20 (0.93–1.47)	1.30 (1.00–1.70)	1.11 (0.92–1.39)	0.116
Dialysis, *n* (%)	2 (0.8)	1 (1.4)	0 (0.0)	1 (1.0)	0.605
NT-proBNP (ng/L), median (IQR)	3,194.0 (1,387.3–7,615.3)	3,853.0 (1,499.0–9,968.0)	3,927.0 (1,559.0–7,778.0)	2,977.5 (1,142.3–6,324.5)	0.607
NYHA stadium III or IV; *n* (%)	195 (76.8)	65 (89.0)	62 (77.5)	68 (67.3)	0.004
Any malignant disease, *n* (%)	44 (17.2)	17 (23.3)	14 (17.5)	13 (12.6)	0.181
Mean transvalvular gradient (mmHg), median (IQR)	30.0 (18.0–39.0)	33.0 (20.0–44.5)	28.0 (18.0–36.0)	29.0 (17.0–38.0)	0.085
Isolated at least moderate aortic valve stenosis, *n* (%)	43 (16.8)	12 (16.4)	11 (13.8)	20 (19.4)	0.593
Isolated at least moderate aortic valve regurgitation, *n* (%)	77 (30.1)	19 (26.0)	25 (31.3)	33 (32.0)	0.667
At least moderate aortic valve stenosis and regurgitation, *n* (%)	127 (49.6)	41 (56.2)	40 (50.0)	46 (44.7)	0.322
Time from index procedure (years), median (IQR)	10.0 (7.0–14.0)	8.0 (6.0–11.0)	11.0 (8.0–14.3)	12.0 (9.0–14.0)	<0.001
Surgical valve type, *n* (%)					
Carpentier-Edwards Perimount, CE-SAV (Edwards Lifesciences, Irvine, CA, USA)	62 (24.2)	16 (21.9)	17 (21.3)	29 (28.2)	0.481
Medtronic Freestyle, Mosaic, Hancock (Medtronic, Minneapolis, MN, USA)	87 (34.0)	21 (28.8)	29 (36.3)	37 (35.9)	0.538
LivaNova Mitroflow (LivaNova, London, United Kingdom)	52 (20.3)	25 (34.2)	19 (23.8)	8 (7.8)	<0.001
Sorin Pericarbon, Freedom Solo (Sorin Group, Milano, Italy)	15 (5.9)	7 (9.6)	4 (5.0)	4 (3.9)	0.262
St Jude Medical Trifecta (St. Jude Medical, St. Paul, MN, USA)	10 (3.9)	1 (1.4)	3 (3.8)	6 (5.8)	0.322
LivaNova Perceval (LivaNova, London, United Kingdom)	10 (3.9)	0 (0.0)	1 (1.3)	9 (8.7))	0.004
Other/unknown	20 (7.8)	3 (4.1)	7 (8.8)	10 (9.7)	0.368
Stented surgical valve prosthesis, *n* (%)	209 (81.6)	64 (87.7)	65 (81.3)	80 (77.7)	0.137
Stentless surgical valve prosthesis, *n* (%)	21 (8.2)	8 (10.9)	6 (7.5)	7 (6.8)	0.220
Sutureless surgical valve prosthesis, *n* (%)	10 (3.9)	0 (0.0)	1 (1.3)	9 (8.7)	0.004
Surgical valve size ≤21 mm, *n* (%)	53 (20.7)	17 (23.3)	19 (23.8)	17 (16.5)	0.487
Surgical valve true ID ≤19 mm, *n* (%)	94 (36.7)	29 (39.7)	30 (37.5)	35 (34.0)	0.298

IQR, interquartile range; BMI, body mass index; NT-proBNP, N-terminal prohormone of brain natriuretic peptide; NYHA, New York Heart Association; ID, inner diameter.

### Procedural data

Procedure time declined over the study period [100.0 (83.0–123.0) vs. 102.5 (79.8–162.0) vs. 68.0 (50.0–105.0) minutes; *p* < 0.001], despite an increase of concomitant procedures (BASILICA, BVF).

Access proportions changed distinctly with an increase in the use of the transfemoral approach (75.3% in period 1, 98.8% in period 2, and 100% in period 3; *p* < 0.001). Correspondingly, the rate of transapical access declined from 17.8% (*n* = 13) in period 1 to 0% (*n* = 0) in later periods (*p* < 0.001).

While in periods 1 and 2, the most commonly used THV consisted of a supra-annular self-expanding (SE) THV, a shift to an intra-annular SE THV was seen in period 3 [5.5% (*n* = 4) vs. 1.3% (*n* = 1) vs. 12.6% (*n* = 13); *p* = 0.010]. The most frequently implanted THVs in the total cohort were Medtronic CoreValve/Evolut/Evolut Pro (Medtronic, Minneapolis, MN, USA) with a rate of 61.3% (*n* = 157), followed by Edwards Sapien/Sapien XT/Sapien 3/Sapien 3 Ultra (Edwards Lifesciences, Irvine, CA, USA) with a rate of 23.4% (*n* = 60), NVT Allegra (NVT, Vancouver, BC, Canada) with an implantation rate of 6.6% (*n* = 17), and Abbott Navitor (Abbott Park, IL, USA) with 5.1% (*n* = 13).

Over time, more BASILICA procedures for the prevention of coronary obstruction were performed (0% vs. 17.5% vs. 19.4%; *p* < 0.001), and rates of valve fracturing steadily increased (0% vs. 6.3% vs. 7.8%; *p* = 0.058).

The utilization of cerebral protection devices witnessed a distinct decline in period 3 (56.2% vs. 68.8% vs. 18.4%; *p* < 0.001).

Detailed procedural data are summarized in Table 2.

**Table 2:** Procedural data.

Variable	All (*n* = 256)	2013–2016 (*n* = 73)	2017–2020 (*n* = 80)	2021–2023 (*n* = 103)	*p*-Value
Procedure time (min), median (IQR)	90.0 (65.0–130.0)	100.0 (83.0–123.0)	102.5 (79.8–162.0)	68.0 (50.0–105.0)	<0.001
Access, *n* (%)					
Transfemoral	237 (92.6)	55 (75.3)	79 (98.8)	103 (100.0)	<0.001
Transapical	13 (5.1)	13 (17.8)	0 (0)	0 (0)	<0.001
Transaxillary	4 (1.6)	3 (4.1)	1 (1.3)	0 (0)	0.092
Transaortic	2 (0.8)	2 (2.7)	0 (0)	0 (0)	0.080
Implanted THV, *n* (%)					
Edwards Sapien/Sapien XT/Sapien 3/Sapien 3 Ultra (Edwards Lifesciences, Irvine, CA, USA)	60 (23.4)	11 (15.1)	14 (17.5)	35 (34.0)	0.005
Medtronic CoreValve/Evolut/Evolut Pro (Medtronic, Minneapolis, MN, USA)	157 (61.3)	54 (74.0)	48 (60.0)	55 (53.4)	0.021
JenaValve (JenaValve, Munich, Germany)	3 (1.2)	3 (4.1)	0 (0)	0 (0)	0.022
SJM Portico (St. Jude Medical, St. Paul, MN, USA)	5 (2.0)	4 (5.5)	1 (1.3)	0 (0)	0.030
Medtronic Engager (Medtronic, Minneapolis, MN, USA)	1 (0.4)	1 (1.4)	0 (0)	0 (0)	0.284
NVT Allegra (NVT, Vancouver, BC, Canada)	17 (6.6)	0 (0)	17 (21.3)	0 (0)	<0.001
Abbott Navitor (Abbott Laboratories, Abbott Park, IL, USA)	13 (5.1)	0 (0)	0 (0)	13 (12.6)	<0.001
SE THV intra-annular, *n* (%)	18 (7.0)	4 (5.5)	1 (1.3)	13 (12.6)	0.010
SE THV supra-annular, *n* (%)	174 (68.0)	54 (74.0)	65 (81.3)	55 (53.4)	<0.001
BE THV intra-annular, *n* (%)	60 (23.4)	11 (15.1)	14 (17.5)	35 (34.0)	0.005
Preballooning, *n* (%)	25 (9.8)	5 (6.8)	6 (7.5)	14 (13.6)	0.237
Postballooning, *n* (%)	129 (50.4)	39 (53.4)	45 (56.3)	45 (43.7)	0.200
Contrast agent (mL), median (IQR)	125.0 (74.5–188.3)	127.0 (93.5–179.0)	137.0 (99.0–199.5)	95.0 (59.0–169.0)	0.002
Use of cerebral protection device, *n* (%)	115 (44.9)	41 (56.2)	55 (68.8)	19 (18.4)	<0.001
Vascular closure system, *n* (%)					
MANTA (Teleflex, Wayne, PA, USA)	118 (47.8)	0 (0.0)	37 (46.8)	81 (82.7)	<0.001
ProGlide (Abbott Laboratories, Abbott Park, IL, USA)	59 (23.7)	17 (23.3)	41 (51.2)	1 (1.0)	<0.001
ProStyle (Abbott Laboratories, Abbott Park, IL, USA)	13 (5.2)	0 (0.0)	0 (0.0)	13 (13.2)	<0.001
ProStar (Abbott Laboratories, Abbott Park, IL, USA)	43 (17.3)	41 (56.9)	1 (1.3)	1 (1.0)	<0.001
BASILICA procedure, *n* (%)	34 (13.3)	0 (0.0)	14 (17.5)	20 (19.4)	<0.001
BVF, *n* (%)	13 (5.1)	0 (0.0)	5 (6.3)	8 (7.8)	0.058

IQR, interquartile range; SE THV, self-expanding transcatheter heart valve; BE THV, balloon-expanding transcatheter heart valve; BASILICA, bioprosthetic or native aortic scallop intentional laceration to prevent iatrogenic coronary artery obstruction during TAVI; BVF, bioprosthetic valve fracturing.

### Outcome

The length of intensive care unit (ICU) stay significantly decreased over time [1.0 (1.0–3.0) vs. 1.0 (1.0–2.0) vs. 1.0 (1.0–1.0) days; *p* < 0.001].

Outcome parameters according to VARC-3, such as myocardial infarction [2.7% (95% CI 0.3%–9.5%) vs. 2.5% (95% CI 0.3%–8.7%) vs. 1.0% (95% CI 0.0%–5.4%); *p* = 0.656], disabling stroke [0.0% (95% CI 0.0%–4.9%) vs. 1.3% (95% CI 0.0%–6.8%) vs. 1.0% (95% CI 0.0%–5.4%); *p* = 0.653], type 3 or 4 bleeding [8.2% (95% CI 3.1–17.0%) vs. 12.5% (95% CI 6.2–21.8%) vs. 4.9% (95% CI 1.6–11.1%); *p* = 0.180], major vascular complications [0.0% (95% CI 0.0%–4.9%) vs. 3.8% (95% CI 0.8–10.6%) vs. 1.9% (95% CI 0.2%–6.7%); *p* = 0.248], permanent pacemaker implantation [5.5% (95% CI 1.5–13.4%) vs. 1.3% (95% CI 0.0%–6.8%) vs. 3.9% (95% CI 1.1%–9.8%); *p* = 0.352], or acute kidney injury stage II or III [4.1% (95% CI 0.9–11.5%) vs. 1.3% (95% CI 0.0%–6.8%) vs. 1.0% (95% CI 0.0%–5.4%); *p* = 0.294], did not show significant changes over time. In one instance, conversion to sternotomy was performed because of a ventricular perforation in a patient undergoing VIV with BASILICA.

The rates of at least moderate paravalvular leakage (PVL) following ViV were stable over time (0 (0.0%) vs. 1 (1.3%) vs. 1 (1.0%); *p* = 0.655). The postprocedural transvalvular mean gradient decreased over time [17.3 (IQR 13.3–22.8) vs. 12.0 (IQR 9.0–18.0) vs. 11.0 (IQR 7.3–17.0) mmHg; *p* < 0.001].

The rates of VARC-3 endpoint device success numerically increased over time without significant differences, from 91.8% [*n* = 67, (95% CI 83.0–96.9%)] in period 1 to over 92.5% [*n* = 74, (95% CI 84.4–97.2%)] in period 2 and to 98.1% [*n* = 101, (95% CI 93.2–99.8%)] in period 3 (*p* = 0.123), because of a lower number of patients with postprocedural transvalvular mean gradients ≥ 20 mmHg. The rate of 30-day mortality declined from 5.5% (95% CI 1.5–13.6%) in period 1 to 0% (95% CI 0.0%–3.5%) in period 3 without any significance.

**Figure 1:**
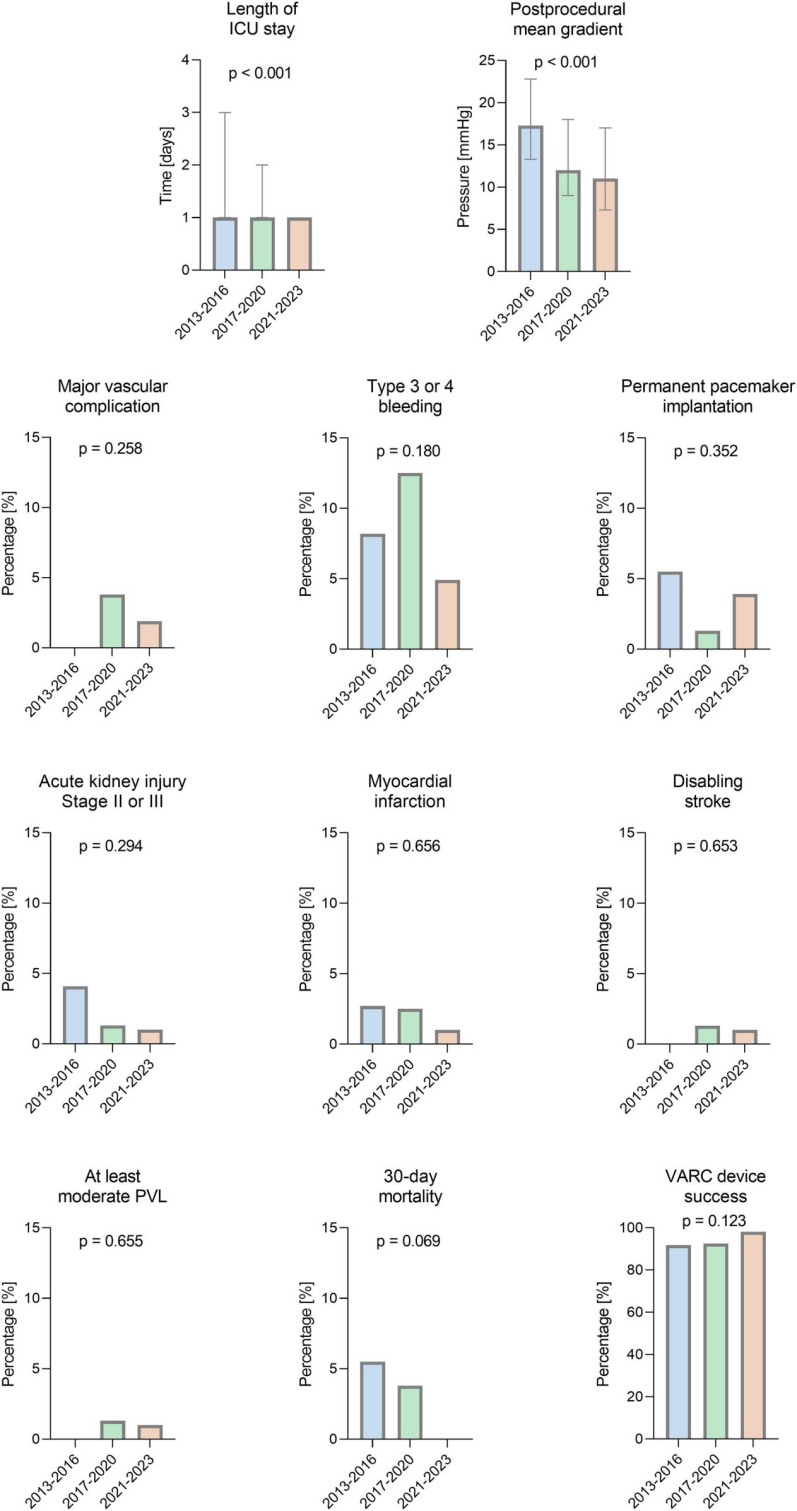
Comparison of outcomes for three time periods of valve-in-valve procedures. VARC-3 endpoints of ViV procedures for degenerated bioprostheses between 2013 and 2023 at our center. PVL, paravalvular leakage; ICU, intensive care unit.

Detailed outcome parameters are given in Table 3. A comparison of outcome parameters for the three time periods of ViV procedures is shown in Figure 1.

**Table 3:** Outcome parameters at 30 days.

Variable	All (*n* = 256)	2013–2016 (*n* = 73)	2017–2020 (*n* = 80)	2021–2023 (*n* = 103)	*p*-Value
NT-proBNP [ng/L], median (IQR)	2,392.5 (849.5–5,546)	5,645.5 (2,158.0–7,105.5)	2,271.0 (763.0–9,162.0)	2,216.0 (849.0–4,456.0)	0.356
Valve malposition, *n* (%)	6 (2.3)	3 (4.1)	1 (1.3)	2 (1.9)	0.476
Pericardial tamponade, *n* (%)	1 (0.4)	0 (0.0)	0 (0.0)	1 (1.0)	0.474
Hemodynamic shock, CPR, *n* (%)	6 (2.3)	0 (0.0)	5 (6.3)	1 (1.0)	0.019
Coronary ostia occlusion, *n* (%)	2 (0.8)	1 (1.4)	1 (1.3)	0 (0.0)	0.506
Aortic root rupture, *n* (%)	0 (0.0)	0 (0.0)	0 (0.0)	0 (0.0)	/
Conversion to CPB, *n* (%)	1 (0.4)	0 (0.0)	0 (0.0)	1 (1.0)	0.474
Length of ICU stay (days), median (IQR)	1.0 (1.0–1.25)	1.0 (1.0–3.0)	1.0 (1.0–2.0)	1.0 (1.0–1.0)	<0.001
Major vascular complication, *n* (%)	5 (2.0)	0 (0.0)	3 (3.8)	2 (1.9)	0.248
Type 3 or 4 bleeding, *n* (%)	21 (8.2)	6 (8.2)	10 (12.5)	5 (4.9)	0.180
Permanent pacemaker implantation, *n* (%)	9 (3.6)	4 (5.5)	1 (1.3)	4 (3.9)	0.352
Acute kidney injury stage II or III, *n* (%)	5 (2.0)	3 (4.1)	1 (1.3)	1 (1.0)	0.294
Myocardial infarction, *n* (%)	5 (2.0)	2 (2.7)	2 (2.5)	1 (1.0)	0.656
Disabling stroke, *n* (%)	2 (0.8)	0 (0.0)	1 (1.3)	1 (1.0)	0.653
Postprocedural mean gradient (mmHg), median (IQR)	14.0 (9.0–19.0)	17.3 (13.3–22.8)	12.0 (9.0–18.0)	11.0 (7.3–17.0)	<0.001
PVL ≥ moderate, *n* (%)	2 (0.8)	0 (0.0)	1 (1.3)	1 (1.0)	0.655
VARC Device Success, *n* (%)	242 (94.5)	67 (91.8)	74 (92.5)	101 (98.1)	0.123
30-day mortality, *n* (%)	7 (2.7)	4 (5.5)	3 (3.8)	0 (0.0)	0.069

IQR, interquartile range; NT-proBNP, N-terminal prohormone of brain natriuretic peptide; CPR, cardiopulmonary resuscitation; ICU, intensive care unit; PVL, paravalvular leakage; VARC, Valve Academic Research Consortium.

Procedural changes within the studied time period were analyzed for their impact on the VARC-3 endpoint “device success.” None of the examined variables (time period, BE THV, SE THV, transfemoral access, cerebral protection, preballooning, postballooning, BASILICA procedure) were identified as an independent factor predicting the endpoint device success.

**Figure 2:**
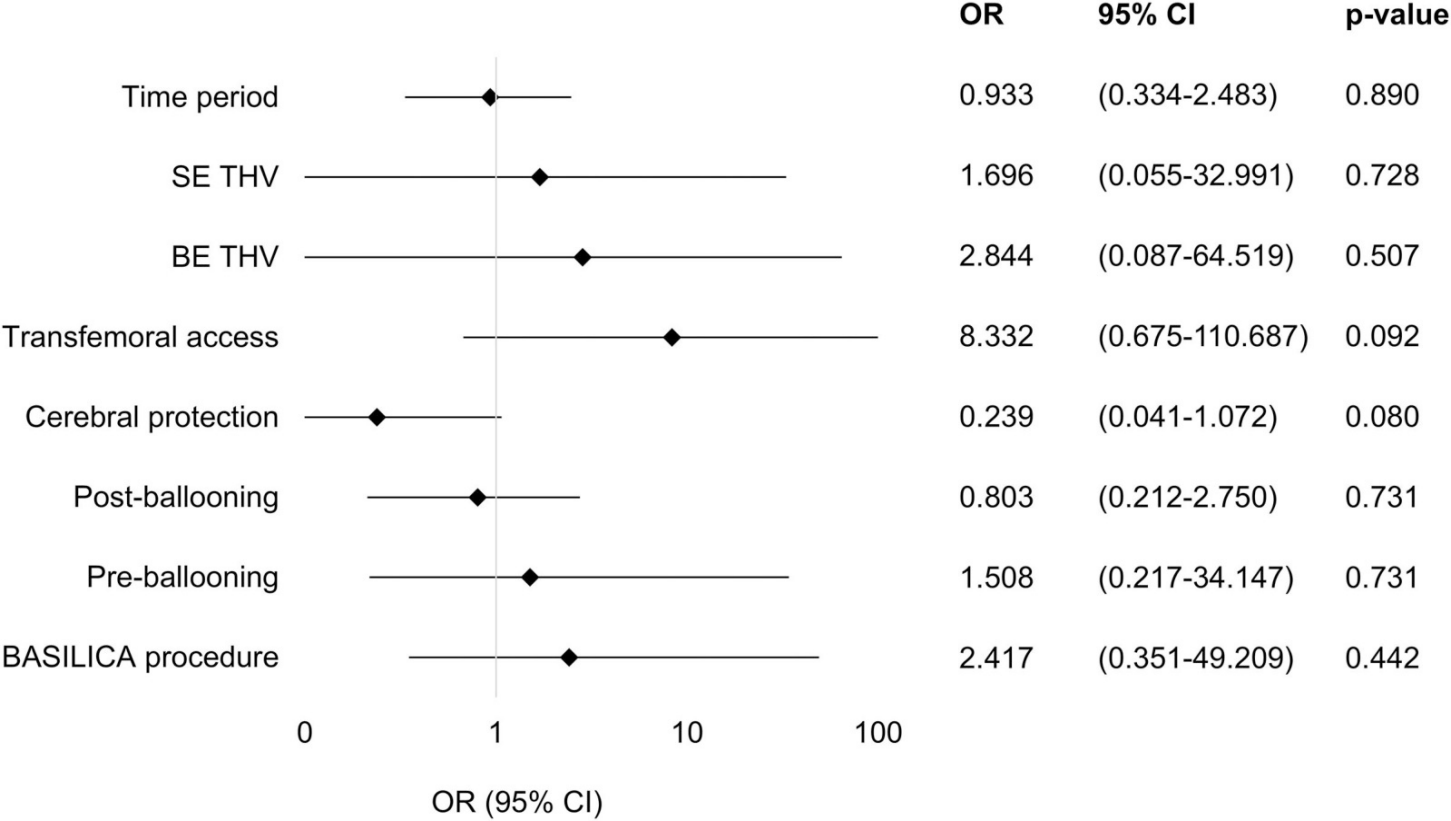
Factors predicting VARC-3 endpoint device success. A forest plot of the multivariate regression model used to identify the factors predicting the VARC-3 composite endpoint device success. Odds ratios and 95% confidence intervals of all tested parameters in the model are shown using a logarithmic *x*-axis. None of the variables included in the model were identified as independent predicting device success. OR, odds ratio; CI, confidence interval; SE THV, self-expanding transcatheter heart valve; BE THV, balloon-expanding transcatheter heart valve; BASILICA, bioprosthetic or native aortic scallop intentional laceration to prevent iatrogenic coronary artery obstruction during TAVI.

A forest plot of the multivariate regression model used to identify the factors predicting the VARC-3 composite endpoint device success is shown in Figure 2.

### Echocardiographic midterm results 12 months after implantation

An echocardiographic follow-up 12 months after ViV determined a mean transvalvular gradient of 12 (8–17) mmHg in the total cohort without significant differences between time periods. There was no transvalvular regurgitation in 96.8% of all patients, and none of the patients presented with a moderate or severe regurgitation.

Symptomatic burden represented by NYHA functional classes showed no significant differences between the patient groups (*p* = 0.366).

Detailed echocardiographic midterm results at 12 months after implantation are presented in Table 4.

**Table 4:** Follow-up transthoracic echocardiography 12 months after implantation.

Variable	All (*n* = 63)	2013–2016 (*n* = 27)	2017–2020 (*n* = 18)	2021–2023 (*n* = 18)	*p*-Value
Mean transvalvular gradient (mmHg), median (IQR)	12 (8–17)	13 (9.5–21)	11 (8.3–12)	9 (6–16.8)	0.124
Left ventricular ejection fraction, *n* (%)					0.007
45–54%	10 (15.9)	1 (3.7)	7 (38.9)	2 (11.1)	
30–44%	8 (12.7)	3 (11.1)	2 (11.1)	3 (16.7)	
<30%	3 (4.8)	0 (0.0)	3 (16.7)	0 (0.0)	
Transvalvular regurgitation, *n* (%)					0.623
None	61 (96.8)	26 (96.3)	17 (94.4)	18 (100.0)	
Trace	2 (3.2)	1 (3.7)	1 (5.6)	0 (0.0)	
Mild	0 (0.0)	0 (0.0)	0 (0.0)	0 (0.0)	
Moderate	0 (0.0)	0 (0.0)	0 (0.0)	0 (0.0)	
Severe	0 (0.0)	0 (0.0)	0 (0.0)	0 (0.0)	
Paravalvular regurgitation, *n* (%)					0.369
None	48 (76.2)	21 (77.8)	12 (66.7)	15 (83.3)	
Trace	7 (11.1)	2 (7.4)	3 (16.7)	2 (11.1)	
Mild	8 (12.7)	4 (14.8)	3 (16.7)	1 (5.6)	
Moderate	0 (0.0)	0 (0.0)	0 (0.0)	0 (0.0)	
Severe	0 (0.0)	0 (0.0)	0 (0.0)	0 (0.0)	
NYHA, *n* (%)					0.366
II	19 (30.2)	7 (25.9)	5 (27.8)	7 (38.9)	
II-III	9 (14.3)	7 (25.9)	2 (11.1)	0 (0.0)	
III	3 (4.8)	1 (3.7)	1 (5.6)	1 (5.6)	
IV	0 (0.0)	0 (0.0)	0 (0.0)	0 (0.0)	

IQR, interquartile range.

## Discussion

This study highlights important temporal shifts in the practice and outcomes of ViV procedures. The main findings of this study are (I) aortic ViV procedures for degenerated bioprostheses showed significant changes in patient characteristics toward lower risk profiles and lower symptomatic burden with stable age over time, (II) advancements in technical approaches such as transfemoral access, BASILICA procedures, and valve fracturing have expanded eligibility for patients previously considered unsuitable for interventional treatment, leading to an increase of treated patients over time and improvement of hemodynamic outcome parameters, (III) the peak incidence of degenerated Mitroflow (LivaNova, London, United Kingdom) prostheses appears to have been reached, (IV) the impact of randomized controlled trials demonstrating the limited efficacy of cerebral protection devices has influenced clinical practice, (V) early outcomes such as device success, stroke, and 30-day mortality were found to be excellent in this study, with improvement over time, and 0% mortality was achieved in period 3, highlighting the clinical efficacy and safety of the ViV procedures.

### Changes in patient characteristics

Patients presented with a significant decrease in risk profiles and a lower symptomatic burden in later time periods. A change in patient age could not be detected. These findings are in line with those of previous work from our group with reference to change in risk profiles and patient age in TAVI for symptomatic native aortic valve stenosis (13) and confirm the influence of randomized controlled trials comparing TAVI and SAVR in low-risk patients on clinical daily practice (NOTION, PARTNER 3, Evolut Low Risk trial) (14–16). Although redo SAVR is a safe therapy modality in contemporary practice (17), its inherently less invasive nature seems to play a crucial role in decision-making in the context of heart team discussions in this rather elderly patient collective.

The threshold for treating patients with ViV declined over time because of encouraging results and enhanced clinical safety over time. Therefore, patients in later time periods were treated earlier with less symptoms and lower-risk patients were treated with ViV because of the improved predictability of hemodynamic results.

Moreover, analyses have shown that the durability of the THV extends up to 10 years, which is similar to that of surgically implanted bioprostheses (18). Because the mean patient age of the investigated patient cohort is above the mean life expectancy of men and slightly below the mean life expectancy of women in Germany, it can be assumed that a majority of patients in the investigated patient cohort who underwent ViV will not outlive the implanted THV.

Within the context of aortic valve stenosis, the relevance of lifetime management remains particularly important for younger patients. Our results suggest that younger patients are still not increasingly undergoing TAVI as ViV procedures in degenerated aortic bioprostheses and rather receive redo SAVR at our center (19). This approach aligns with the current concepts of lifetime valve management, emphasizing individualized treatment strategies based on patient age and clinical risk. In younger patients with acceptable surgical risk, redo SAVR remains the preferred option to optimize long-term durability. With increasing risk profiles, ViV TAVI provides a less invasive alternative with favorable safety outcomes. However, in patients for whom a third intervention may be anticipated, the use of intra-annular transcatheter valves should be considered to facilitate a potential second ViV procedure in the future.

### Periprocedural techniques

Over the last few years, reduced sheath sizes and improvements in delivery systems have made transfemoral access applicable to a higher number of patients. Consequently, the significance of alternative access routes, which are associated with increased perioperative mortality and morbidity, has declined (20). In this study, a shift toward transfemoral access was seen, with 100% transfemoral access in contemporary practice, accompanied by a shift to a second access via the radial artery. These modalities certainly contributed to the safety of ViV, with a decline, although not significant, in bleeding and vascular complications (21).

Over time, a significant increase of concomitant BASILICA and BVF procedures was seen in our ViV cohort. Most likely, these concomitant procedures contributed to the herein seen increase of patient numbers by expanding approachable anatomies for ViV. Specifically, patients with bioprostheses with externally mounted leaflets (MitroFlow, Trifecta), shallow sinuses of Valsalva, or a low valve to coronary distance were commonly considered unsuitable for ViV. However, with the implementation of BASILICA, ViV became applicable to those patients without a significant increase in perioperative risk (22). Furthermore, an increase in device success rates was seen accompanied by a steady decrease in postoperative transvalvular pressure gradients, which is most probably a consequence of higher rates of BVF in later time periods, sophisticated implant techniques using the cusp-overlap technique, and alignment of the proximal THV stent at the highest point of the bioprosthetic stent.

The cusp-overlap technique, initially developed for native TAVI with SE THV to optimize implantation height and reduce PVL and pacemaker rates, is of lesser relevance in ViV procedures. In ViV using SE THV, however, it may assist in accurate positioning by aligning the THV stent frame with the surgical bioprosthesis (“stent-on-stent”). For BE THV, cusp-overlap views are generally not necessary, as the radiopaque surgical stent frame provides sufficient fluoroscopic guidance during deployment.

Although not all bioprosthetic stents are fracturable, it has been shown that BVF leads to improved hemodynamics after ViV (23). Furthermore, the cusp-overlap technique used in TAVI for severe symptomatic aortic valve stenosis has been shown to improve the prediction of implantation height, leading to a subsequent reduction in PVL and permanent pacemaker implantation rates (24).

In several studies, implantation of SE THV in degenerated bioprostheses was associated with lower postprocedural transvalvular gradients compared with BE THV (25, 26). A subanalysis of the VIVID registry identified intra-annular THV design as an independent predictor for elevated postprocedural gradients (5). The rate of survival following SE or BE THV for ViV procedures was found to be similar (25). In this study, preferred implanted valves consisted of SE THV. However, the proportion of BE THV increased over time. Early mortality rates of patients did not change significantly over time. In the 12-month echocardiographic follow-up, no significant difference in the transvalvular gradients was observed across different time periods.

Although the proportion of small surgical bioprostheses remained unchanged over time, the persistently high device success rate observed in this cohort may reflect the early adoption of supra-annular SE THV in small surgical valves and a cautious patient selection strategy. These factors likely contributed to favorable postprocedural hemodynamics, even before BVF and other adjunctive techniques became routinely implemented.

### Index bioprostheses

Bioprostheses with externally mounted leaflets were shown to have a higher incidence of early structural valve deterioration leading to higher reoperation rates when compared with bioprostheses with internally mounted leaflets. Furthermore, these valves demonstrated a higher all-cause mortality (27). In this study, we observed a peak incidence of degenerated bioprostheses with externally mounted leaflets in period 1. The decline of ViV for these specific valve types in later time periods may be explained by a reduced implantation rate as a direct consequence of these findings. Furthermore, an increase of sutureless valves treated by ViV was seen, a finding that is worth further observation.

### Cerebral protection

Several randomized controlled trials have demonstrated the limited efficacy of cerebral protection devices in TAVI (28–31). In our study, a direct impact of these findings on clinical practice in later time periods was seen without compromising patient safety in terms of postprocedural stroke.

While the utilization of cerebral embolic protection devices has significantly decreased over time in our cohort, it remains higher than in recent randomized controlled trials investigating TAVI for severe symptomatic aortic valve stenosis in contemporary practice (DEDICATE trial) (32).

### Clinical outcomes

In this cohort, excellent 30-day clinical outcomes with a device success rate of 98.1% and a mortality rate of 0% in time period 3 without significant changes over time were detected. Procedure duration presented with a significant decrease over time. ICU stay decreased significantly, mainly because of reduced ICU times in contemporary TAVI practice. At 12 months after ViV, similar transvalvular mean gradients and rates of PVL were described in echocardiographic follow-up.

Compared with a meta-analysis of 5,500 patients treated with ViV procedures, rates of 30-day mortality, stroke, and permanent pacemaker implantation are lower in this analysis, highlighting the procedures’ safety (33). Data from the Valve-in-Valve International Data (VIVID) registry showed a 30-day mortality rate of 5.3% in 1,550 patients from 110 centers (34), and results from this analysis presented a 30-day mortality rate of 0% in latest time periods. As procedural experience increased and device technology improved, ViV implantation was offered to patients with lower baseline risk and fewer symptoms. This evolution probably contributed to the overall stability of adverse event rates, including stroke, pacemaker implantation, and mortality. Moreover, the lower complication rates may reflect both enhanced operator expertise and more refined patient selection in the later study period.

Compared with redo SAVR, ViV is associated with lower incidences of periprocedural complications (35), whereas the incidence of early mortality after ViV and redo SAVR is similar (35, 36). However, ViV is commonly considered to present significantly higher incidences of paravalvular leakage and increased mean transvalvular gradients compared with redo SAVR, and previously identified risk factors for adverse outcomes in ViV in terms of mortality are small bioprosthetic valves, age, and non-transfemoral access (36–38), emphasizing the need for a thorough heart team discussion of every individual patient with a deteriorated surgical bioprosthesis in order to determine the best treatment modality in the context of aortic valve lifetime management. Particularly, younger patients who most probably will outlive the implanted THV may benefit from redo SAVR with aortic annulus enlargement (39) to facilitate future ViV procedures.

### Study limitations

The present study is limited by its retrospective, single-center design, which may affect the generalizability of the results. Patients were not randomized to a specific treatment strategy or time point, and potential selection bias with unmeasured confounders cannot be ruled out. In addition, the absence of a comparator group, such as patients undergoing redo surgical aortic valve replacement, precludes a direct comparison of outcomes between treatment modalities.

## Conclusions

This 10-year study of aortic ViV procedures for degenerated bioprostheses showed significant changes in patient risk profile and procedural measures over time. Advancements in technical approaches such as transfemoral access, BASILICA procedures, and BVF have expanded eligibility for patients previously considered unsuitable for interventional treatment. In addition, the peak incidence of degenerated Mitroflow prostheses appears to have been reached. Moreover, the impact of randomized controlled trials demonstrating the limited efficacy of cerebral protection devices has influenced clinical practice. In this study, it was found that early outcomes such as device success, stroke, and 30-day mortality were excellent, with improvement over time, highlighting the clinical efficacy and safety of the ViV procedures.

## Data Availability

The raw data supporting the conclusions of this article will be made available by the authors without undue reservation.
